# A novel flow cytometric assay for measurement of *In Vivo *pulmonary neutrophil phagocytosis

**DOI:** 10.1186/1471-2180-6-61

**Published:** 2006-07-12

**Authors:** Elizabeth A Vander Top, Greg A Perry, Martha J Gentry-Nielsen

**Affiliations:** 1 Department of Medical Microbiology and Immunology, Creighton University School of Medicine, Omaha, Nebraska, USA; 2 Research Service, Omaha Veterans Affairs Medical Center, Omaha, Nebraska, USA

## Abstract

**Background:**

Phagocytosis assays are traditionally performed *in vitro *using polymorphonuclear leukocytes (PMNs) isolated from peripheral blood or the peritoneum and heat-killed, pre-opsonized organisms. These assays may not adequately mimic the environment within the infected lung. Our laboratory therefore has developed a flow cytometric *in vivo *phagocytosis assay that enables quantification of PMN phagocytosis of viable bacteria within the lungs of rats. In these studies, rats are injected transtracheally with lipopolysaccharide (LPS) to recruit PMNs to their lungs. They are then infected with live 5(-and 6) carboxyfluorescein diacetate succinimidyl ester (CFDA/SE) labeled type 3 *Streptococcus pneumoniae*. Bronchoalveolar lavage is performed and resident alveolar macrophages and recruited PMNs are labeled with monoclonal antibodies specific for surface epitopes on each cell type. Three color flow cytometry is utilized to identify the cell types, quantify recruitment, and determine uptake of the labeled bacteria.

**Results:**

The viability of the alveolar macrophages and PMNs isolated from the lavage fluid was >95%. The values of the percentage of PMNs in the lavage fluid as well as the percentage of PMNs associated with CFSE-labeled *S. pneumoniae *as measured through flow cytometry showed a high degree of correlation with the results from manual counting of cytospin slides.

**Conclusion:**

This assay is suitable for measuring bacterial uptake within the infected lung. It can be adapted for use with other organisms and/or animal model systems.

## Introduction

Phagocytosis and killing of pathogens by resident alveolar macrophages and recruited polymorphonuclear leuckocytes (PMNs) is integral in clearing bacterial respiratory tract infections. Because alveolar macrophages (AM) do not efficiently phagocytose most strains of *Streptococcus pneumoniae *(the pneumococcus), uptake and killing by recruited PMNs is particularly important during pneumococcal pneumonia [1]. For a number of years, our laboratory has been studying the effects of ethanol ingestion and liver disease on pulmonary PMN function. We began with traditional phagocytosis assays performed *in vitro *with peripheral or peritoneal PMNs and heat-killed, pre-opsonized organisms. Such assays are limited, however, in that they cannot adequately mimic the conditions present within the infected lung. Therefore, they may not detect all host defects leading to reduced phagocytic uptake of bacteria during pulmonary infections.

One particular difficulty with the use of *in vitro *assays for measurement of pneumococcal phagocytosis is that the organisms are not taken up unless they are pre-opsonized with serum containing active complement components, even though the serum may contain high titers of specific antipneumococcal antibodies [2]. This need for pre-opsonization of the organisms makes it impossible to examine certain underlying host immune defects such as complement deficiencies. The serotype 3 pneumococcal strain used in our studies (ATCC 6303) presents an additional unique difficulty when used in *in vitro *phagocytosis assays because neither human nor rat PMNs phagocytose this highly encapsulated *S. pneumoniae *strain *in vitro *[2-4]. The type 3 strain's ability to avoid phagocytosis *in vitro *has been hypothesized to be the result of its large capsule that precludes accessibility of its complement and immunoglobulin receptors [3].

Several years ago our laboratory developed an *in vivo *phagocytosis assay that utilized flow cytometry and type 3 pneumococci labeled with Lucifer Yellow [2]. Although a major improvement, this assay still was not physiologically relevant to the infection process because Lucifer Yellow is excluded by live bacteria. This necessitated heat-killing of the bacteria, a process that can alter the pathogen's surface and impair the function of its anti-phagocytic capsule. To overcome this limitation of our original *in vivo *assay, we now have created an *in vivo *phagocytosis assay that utilizes live type 3 pneumococci and 3-color flow cytometry. This assay allows for the measurement of phagocytosis of live, non-opsonized *S. pneumoniae *within the natural environment of the lung, enabling us to effectively mimic the events that occur during an actual pneumococcal infection. This manuscript describes and validates our assay and demonstrates its potential for adaptation to other microorganisms and animal models of infection.

## Results

### Fluorescent labeling of bacteria and bacterial viability

**Figure 1:**
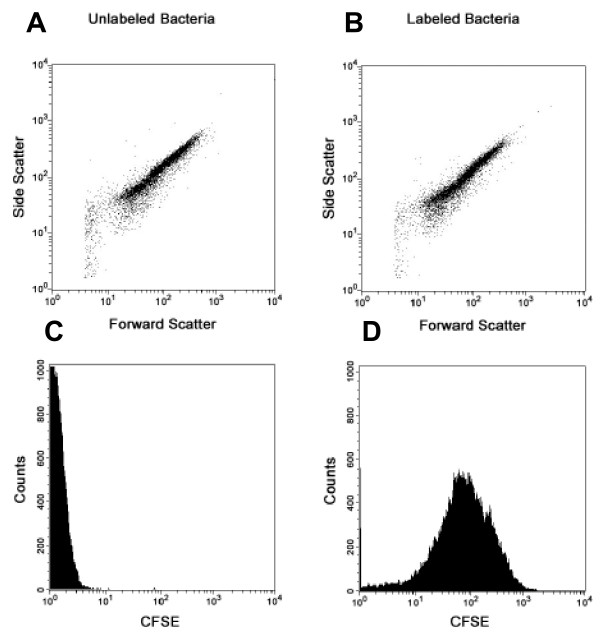
Staining of *S. pneumoniae *with CFDA/SE. Forward vs. side scatter plots of Unlabeled (A) and CFDA/SE labeled (B) type 3 *Streptococcus pneumoniae *are indistinguishable. Histograms of cell counts vs. CFSE staining (the fluorescent intracellular form of the dye), however, clearly demonstrate the shift in fluorescence between unlabeled (C) and CFDA/SE labeled (D) pneumococci.

**Figure 2:**
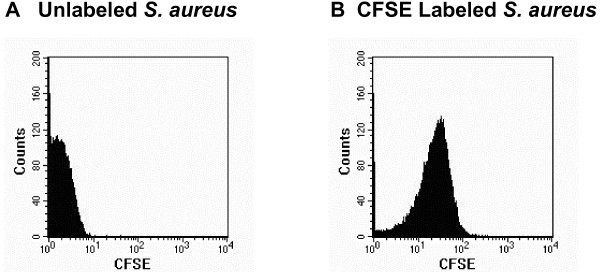
Staining of *S. aureus *with CFDA/SE. Histograms of cell counts vs. CFSE staining, note the shift in fluorescence between unlabeled (A) and CFDA/SE labeled (B) *S. aureus*.

To confirm that the *S. pneumoniae *organisms were adequately labeled, flow cytometry was performed. Figure 1 shows that the bacteria effectively took up the CFDA/SE and fluoresced brightly. To determine if our assay could be utilized for the measurement of *in vivo *phagocytosis of other bacterial organisms, we attempted to CFSE label *Escherichia coli *and *Staphylococcus aureus *as well. Using the protocol described in this paper, CFSE did not effectively label *E. coli *organisms (data not shown). On the other hand, *S. aureus *was labeled by CFSE, although the organisms fluoresced at nearly a log lower intensity than *S. pneumoniae *organisms (Figure 2). The CFSE staining protocol did not affect the viability of any of the organisms (Table 1).

### Flow cytometric analysis of BAL cells

**Figure 3:**
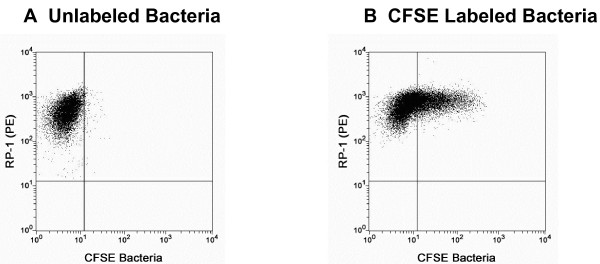
Determination of percentage of PMNs with internalized bacteria. For each day of the assay, unlabeled *S. pneumoniae *were instilled into the lungs of a single rat. The PMNs collected from that rat by BAL [the RP-1 (PE) positive cells] were analyzed for autofluorescence in the CSFE channel and used to set a marker (shown in A) to delineate the PMNs in all other samples that had phagocytosed CFSE-labeled bacteria (shown in B).

To exclude autofluorescence of the PMN and macrophage populations, a sample of BAL cells from a control animal infected with live, but non-fluorescent *S. pneumoniae *was used to calibrate the machine each day the assay was run. Figure 3A shows the PMN population from one representative control animal. Figure 3B is a representative dot plot of a test animal infected with CFSE-labeled pneumococci. The percentage of RP-1-positive cells (PMNs, Figure 3A) or 1C7-positive cells (macrophages, data not shown) that had taken up the CFSE-labeled pneumococci was then determined (Figure 3B).

### Confocal microscopy of BAL cells

**Figure 4:**
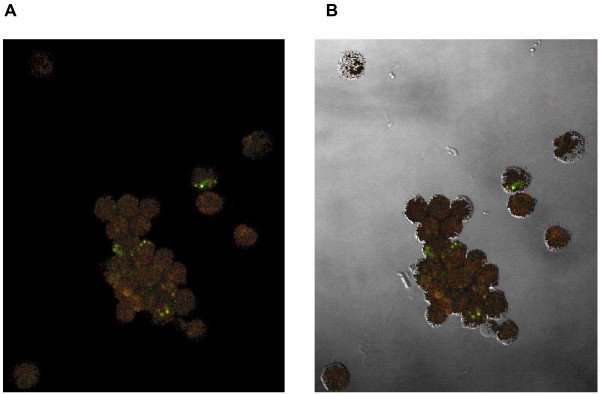
Phase contrast microscopy (A) was used to demonstrate the presence of the bacteria within the pulmonary cells and an overlay technique (B) was used to show that the bacteria were internalized.

Confocal microscopy was performed to ensure that the pneumococci were phagocytosed by the recruited PMNs, not just adherent to the cell surface. Figure 4 shows the confocal (4A) and phase overlay (4B) images of internalized pneumococci.

### Stability of CFSE labeling of pneumococci

**Figure 5:**
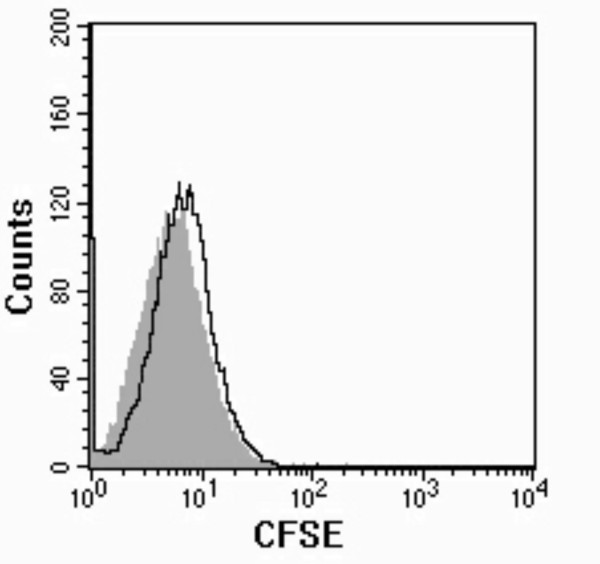
Stability of CFSE Labeling of Pneumococci. To ensure that CFSE did not leak out of labeled pneumococci when they were in the rats' lungs, CFSE labeled pneumococci were incubated with fresh lavage fluid. After centrifugation to remove the bacteria the supernatant was incubated with lavage cells. Lavage cells before incubation with the supernatant (gray shading) and after (black line).

To ensure that our results were not due to release of the CFSE label during incubation of the pneumococci in the rats' lungs and subsequent uptake of CFSE by the PMNs, CFSE-labeled pneumococci were incubated at 37°C for 1 h in fresh BAL fluid. The bacteria were then removed by centrifugation and the resulting supernatant was incubated with BAL cells. Figure 5 shows an overlay image of the lavage cells before (gray shading) and after (black line) incubation with the supernatant showing a negligible shift in the cells' fluorescence.

### Verification by light microscopy of PMN recruitment values

To validate our flow cytometric assay, we compared manual counts of cytospin slides to the flow cytometric data. Viability of the collected BAL cells was consistently >95%. Cytospin preparations stained with Protocol Hema-3 were used to classify 200 cells in each sample based on their morphological characteristics. The percentages of macrophages and PMNs counted on the microscope slides were quite similar to those determined by flow cytometry. A comparison of the PMN values for ten representative animals is shown in Table 2. For all 31 rats examined, the mean percent ± S.E.M. PMN recruitment determined by flow cytometry was 70.9 ± 2.5, whereas the mean determined by manual counts was 66.6 ± 1.9. The correlation coefficient between the values measured by the two methods was 0.488 (*p *< 0.006). The percentage of macrophages present in each sample also correlated well between the two methods of measurement with a correlation coefficient of 0.578 (*p *< 0.0001).

### Verification of phagocytosis values by light microscopy

Manual counts of cytospin slides prepared from each rat also were used to verify the percentage of macrophage and PMN phagocytosis measured by flow cytometry. A minimum of 200 cells were counted in the light microscope to quantify the percentage of each cell population that contained internalized *S. pneumoniae *organisms. Flow cytometry vs. light microscopy PMN phagocytosis values from ten representative samples are shown in Table 3. For all 31 rats examined, the mean percent ± S.E.M. PMN phagocytosis determined by flow cytometry was 16.1 ± 1.4, whereas the mean determined by manual counts was 18.7 ± 1.7. The correlation coefficient for the values determined by the two methods was 0.688 (*p *< 0.0002). Contrary to the values for PMNs, the percentage of phagocytosis values for macrophages did not correlate significantly with a correlation coefficient of 0.0801 (*p *= .319) and values of 3.3 ± 0.4 determined by light microscopy and 7.3 ± 1.0 by flow cytometry.

## Discussion

This manuscript describes an immunological assay to measure PMN recruitment and phagocytosis of viable type 3 *Streptococcus pneumoniae *within the lungs of rats rather than the artificial environment used for *in vitro *assays. Performing the experiment within the rats' lungs allows the simultaneous measurement of recruitment and phagocytosis and precludes the necessity for pre-opsonization of the organisms. One can therefore examine host defects in opsonic capacity as well as phagocyte function. The ability to utilize live bacteria is a major improvement over our earlier *in vivo *assay [2] because it allows for quantification of phagocytic uptake of organisms that are physiologically relevant to those found during an actual pulmonary infection.

One early difficulty in development of the assay was finding a dye that could enter live *S. pneumoniae *and then remain stable within the bacteria rather than leaking out where it could be taken up by the phagocytes to give false positive readings. The dye CFDA/SE fulfilled these requirements; in its' nonfluorescent form it readily enters live bacteria where the acetate groups are cleaved by cytosolic esterases [5]. The CFSE molecule is stable within the bacterial cell, and does not leak out in appreciable amounts, even when the pneumococci are allowed to divide.

Our CFSE staining protocol effectively labeled *S. aureus *organisms, but not *E. coli*. It may be that the gram negative organism is either less permeable to the CFSE molecule or able to pump it out of the cytoplasm (as is evidenced with certain antibiotics). Therefore, the described *in vivo *phagocytosis assay may only be useful for certain organisms and not others. However, alternative fluorescent dyes may be substituted for CFSE in our assay.

As expected, few alveolar macrophages were shown to internalize pneumococci when examined by light microscopy. Unlike the PMN phagocytosis values, there was no agreement or even correlation between the flow cytometric and light microscopy values for the alveolar macrophage population. The reason for the artificially high phagocytosis values by flow cytometry is the intrinsically high autofluorescence of rat macrophage populations [6]. This autofluorescence is at the same wavelength as the CFSE fluorescence, thus precluding accurate measurement of the macrophage phagocytosis. Pneumococci in general and the type 3 strain in particular are known to resist phagocytosis by macrophages [1]. For this reason we optimized our assay for measuring recruitment and phagocytosis of the PMN rather than the macrophage. In order to utilize this assay for the measurement of macrophage phagocytosis of other bacterial strains, it will be important to make several adjustments to this protocol, which may include using an alternate fluorochrome to label the organism and/or a different anti-macrophage antibody.

The high degree of correlation between PMN phagocytosis as determined by flow cytometry and the much more subjective and labor intensive manual counting of cytospin slides validates the flow cytometric method. As with all phagocytosis assays, however, there is concern about the ability to differentiate ingested organisms from those bound to the outside of the phagocytes [7-9]. Traditionally, differentiation of bound but not internalized organisms has been accomplished through the addition of a quenching agent such as ethidium bromide or trypan blue that eliminates fluorescence of bound, but not internalized organisms [7,8,10]. In our assay, using these agents to quench externally bound organisms is not an option because live *S. pneumoniae *exclude these quenching agents. In addition, the monoclonal antibodies used to identify the PMN and macrophage populations bind to surface epitopes on the cells, and the addition of a quenching agent may remove this fluorescence as well. We therefore utilized confocal microscopy to confirm that the organisms are located within the cell membrane rather than bound to the surface of the cells.

## Conclusion

The novel *in vivo *phagocytosis assay described in this manuscript provides a unique opportunity to "visualize" what is occurring within the lungs during an actual infection and to measure several aspects of host defense against pulmonary pathogens. Although the method has been optimized for use with pneumococcal phagocytosis by PMNs in rat lungs, the methods described can well be adapted to measure pulmonary uptake of other microorganisms in alternative animal models as well.

## Methods

### Animals

Male Sprague-Dawley rats (Charles River Laboratories, Kingston, NY) weighing 350–400 g were used. All experimental procedures described herein were approved by the Animal Studies Subcommittee, both at Creighton University and the Omaha Veterans Affairs Medical Center.

### *Streptococcus pneumoniae *growth and labeling conditions

The organism used for the phagocytosis assay was a type 3 *S. pneumoniae *(ATCC 6303; American Type Culture Collection, Rockville, Maryland, USA) that was originally a clinical isolate. It was stored at -80°C in Todd-Hewitt Broth containing 10% glycerol. Before each experiment, the bacteria were grown to mid-log phase in Todd-Hewitt broth supplemented with 5% heat-inactivated rabbit serum (Nieffengger Company, Woodland, CA). They were collected by centrifugation at 10,000 × *g *for 10 minutes, washed twice with phosphate buffered saline (PBS), and resuspended in PBS to an optical density of 1.0 at 540 nm. An aliquot of the suspension then was incubated at 37°C for 30 min in the dark with an equal volume of a 2.0 μM solution of 5(-and 6) carboxyfluorescein diacetate succinimidyl ester (CFDA/SE, Molecular Probes, Eugene, Oregon), the non-fluorescent diacetate form of CFDA that easily passes through cell membranes to label viable bacteria [5]. Once inside the organisms, the molecule is converted to the fluorescent form known as CFSE [5(-and 6) carboxyfluorescein succinimidyl ester].

After washing three times with PBS to remove excess dye, the bacteria were once again resuspended in PBS to an optical density of 2.5 at 540 nm (equivalent to 3 × 10^9 ^colony forming units (cfu)/ml). To ensure that viability of the pneumococci was not altered by CFSE labeling, aliquots of a suspension of *S. pneumoniae *organisms (O.D. 1.0 at 540 nm) were serially diluted on sheep blood agar plates before and after staining.

### Flow cytometry of bacteria

Flow cytometry of bacteria was performed using standard methods for bacterial analysis by flow cytometry. Briefly, forward scatter, side scatter, and all fluorescence parameters were collected in log mode. Instrument thresholding was performed on either side and/or forward scatter signals. CFSE fluorescence was detected on the FACSCalibur (Becton Dickinson, San Jose, CA) using a 530/30 bandpass filter.

### Labeling of other bacterial strains

*Escherichia coli *(ATCC25922) and *Staphylococcus aureus *(ATCC25923) were grown overnight on blood agar plates, and then resuspended in PBS to an optical density of 1.0 at 540 nm. An aliquot of the bacterial suspensions were plated on sheep's blood agar plates to enumerate the colony forming units before staining. Equal volumes of each bacterial strain were incubated with CFSE as described above for *S. pneumoniae*. The bacterial strains were then washed twice with PBS and resuspended to an optical density of 1.0 at 540 nm. An aliquot of the bacterial suspensions were once again plated to determine the effect of CFSE labeling on bacterial viability. Flow cytometry was performed on fixed samples of the *E. coli *and *S. aureus *organisms as described above.

### Pulmonary PMN recruitment and bacterial instillation

PMNs were recruited to the rats' lungs by transtracheal instillation of 20 μg *Escherichia coli *026:B6 lipopolysaccharide (Sigma, St. Louis, MO) in 0.2 mL PBS. This amount of LPS has been shown to consistently increase the percentage of PMN recoverable by bronchoalveolar lavage (BAL) from <2% to >50% (2). Briefly, the rats were anesthetized with isofluorane (Halocarbon Laboratories, River Edge, New Jersey, USA), and blunt dissection to the trachea was performed. A 20 gauge catheter was inserted into the trachea, through which the LPS solution was instilled into each rat's lungs. The skin then was closed around the incision with a small metal clip. Five hours after the LPS instillation, the clip was removed and the rats were infected transtracheally through the same incision with 0.3 mL of the CFDA/SE labeled *S. pneumoniae *(approximately 1 × 10^9 ^cfu/rat). This was followed by instillation of 0.3 ml of air to simulate aspiration. This was followed by instillation of 0.3 ml of air to simulate aspiration. Plate counts of the bacterial suspension were performed immediately before infection, to provide an accurate measure of the organisms' viability.

### Recovery of pulmonary cells

Exactly 15 minutes post-infection, each rat was euthanized with an intraperitoneal injection of 75 mg/kg body weight of pentobarbital (Nembutal, Abbot Laboratories, Abbott Park, Illinois, USA). This short infection period mimics that used in standard *in vitro *phagocytosis assays, and prevents the division of fluorescent pneumococci within the rats' lungs, which would result in a loss of fluorescence intensity. Pulmonary cells were collected by *ex vivo *BAL using a method described previously [11]. Briefly, the rats were exsanguinated by cardiac puncture and their lungs were perfused with 30 ml of PBS to remove contaminating peripheral blood cells. The lungs and trachea then were removed *en bloc*, and pulmonary cells were washed from the lungs by repeated instillation of 10-ml aliquots of ice-cold Hanks' Balanced Salt Solution without Mg^++ ^or Ca^++ ^(HBSS, Gibco/Invitrogen, Carlsbad, CA) through a catheter inserted into the original incision and collected by dependent drainage until a total of 50 ml of lavage fluid was obtained. BAL cells collected from the lavage fluid were washed twice with HBSS using differential centrifugation (180 × g for 8 minutes) to remove unassociated bacteria. Erythrocytes were lysed hypotonically by the addition of 10 ml of deionized water followed in 10 seconds by an equal volume of double strength PBS. The remaining cells were centrifuged and resuspended in PBS containing 4% heat-inactivated fetal calf serum (PBS-FCS), counted on a hemacytometer, and adjusted to a concentration of 2 × 10^7 ^cells/ml. Viability of the cells was assessed through trypan blue exclusion, and aliquots of each sample were reserved for cytocentrifugation and microscopic analysis.

### Flow cytometry

For flow cytometric analysis, 50 μL of cell suspension from each BAL sample (1 × 10^6 ^cells) were stained for 30 minutes in the dark at 4°C in 96-well U-bottom microplates with 50 μL of a monoclonal antibody cocktail containing the phycoerythrin (PE)-conjugated anti-rat granulocyte antibody RP-1 (Pharmingen, San Diego, CA) and the biotinylated anti-rat monocyte antibody 1C7 (Pharmingen). The cells then were washed three times with PBS-FCS and incubated for an additional 5 minutes in the dark at 4°C with allophycocyanin (APC) -conjugated Streptavidin (Pharmingen) to stain the monocytes. After three more washes in PBS-FCS, the BAL cells were resuspended in PBS containing 1% formalin. Negative control samples for each rat consisted of unstained BAL cells and BAL cells stained only with APC-Streptavidin. Three color flow cytometric analysis was performed using a FACSCalibur flow cytometer with dual laser excitation (488 nm and 633 nm). A single sample of BAL cells collected from a rat infected with nonfluorescent type 3 *S. pneumoniae *organisms was included each day to exclude autofluorescence of the RP-1 (PE) positive PMN and 1C7 (APC) positive macrophages populations in the CFSE channel. We also used a gate to identify single PMNs and remove debris, non-cellular and on-axis events. The percentage of RP-1 or 1C7- positive cells then was counted as the percentage of PMNs or macrophages associated with bacteria.

Based on scatter characteristics that included PMNs and macrophages, a minimum of 20,000 cells from each sample were analyzed. The frequency of RP-1 positive PMNs and 1C7-positive macrophages, as well as the percentage of each cell type containing associated fluorescent bacteria was determined for each sample using the CellQuest Pro software (Becton Dickinson, San Jose, CA).

### Microscopic analysis

Cytospin slides (Cytospin 2, Shandon, Cheshire, UK) prepared from each BAL sample analyzed by flow cytometry were stained with Protocol Hema-3 (Fisher Diagnostics, Middletown, Virginia, USA), and 200 cells from each sample were counted manually in a Nikon Labopho-2 (Melville, NY) to verify the percentage of PMNs and macrophages in the BAL cell population and the percentage of each cell type that had phagocytosed *S. pneumoniae*. For confocal microscopy, unstained cytospin slides were prepared of cell samples after they had been treated with the fluorochrome-labeled RP-1 and 1C7 antibodies. The slides were examined with a Zeiss LSM 410 confocal laser scanning microscope with an Argon/Krypton (488 nm/568 nm/647 nm) laser (Goettinger, Germany) to demonstrate that cell-associated bacteria were internalized.

### Analysis of CFSE leakage during pneumococcal division

To determine if the CFSE label would leak out of the pneumococci during bacterial cell division, a 300 μl aliquot of the stained organisms was incubated with an equal volume of fresh rat lavage fluid at 37°C for 1 hour, to mimic the actual infection process. The pneumococci were then removed by centrifugation at 10,000 × g for 10 minutes. The resulting supernatant was incubated with 1 × 10^7 ^BAL cells from the same animal for 30 minutes at 37°C. The BAL cells were washed, fixed and flow cytometry was performed to detect any shift in fluorescence that could be attributable to leakage of CFSE during incubation in the lavage fluid and subsequent staining of the lavage cells.

### Statistical analyses

Normally distributed recruitment and phagocytosis data from microscopic and flow cytometric analyses were compared by Student's *t*-test (SigmaStat 3.0, Leesburg, VA). Non-parametric data were analyzed by the Mann-Whitney rank sum test (SigmaStat 3.0). Agreement between the flow cytometric and manual methods of measurement was quantified using Spearman's rank correlation coefficient (SigmaStat 3.0). A *p *< 0.05 was considered significant for all comparisons.

## Authors' contributions

All authors contributed to the development of the flow cytometric assay. EVT was responsible for all the sample collection, and GP performed all the flow cytometric analysis. EVT and MGN drafted the manuscript. All authors read and approved the final manuscript.

## Figures and Tables

**Table 1:** Effect of CFSE on Bacterial Viability

	*Colony forming units/ml at O.D. of 1.0 at 540 nm*	
Bacterial Strains	Unstained	Stained

*Streptococcus pneumoniae*	7.0 × 10^8^	7.9 × 10^8^
*Staphylococcus aureus*	2.3 × 10^8^	1.9 × 10^8^
*Escherichia coli*	4.9 × 10^8^	4.2 × 10^8^

**Table 2:** Comparison of PMN Recruitment Values Measured by the Two Methods in Ten Representative Samples

	*Percentage of PMNs in BALF*	
Rat	Flow Cytometry	Manual Counts

1	60.8	51.0
2	75.0	70.0
3	57.6	59.0
4	66.3	67.0
5	74.5	71.0
6	62.0	62.0
7	91.3	84.0
8	80.6	81.0
9	71.0	76.0
10	75.1	72.0

**Table 3:** Comparison of PMN Phagocytosis Values Measured by the Two Methods in Ten Representative Samples

	*Percentage of PMNs Containing S. pneumoniae*	
Rat	Flow Cytometry	Manual Counts

1	29.6	27.0
2	20.1	18.0
3	28.5	38.0
4	17.9	27.0
5	14.3	14.0
6	12.6	9.0
7	2.3	5.0
8	7.1	8.0
9	8.4	8.0
10	15.4	19.0
